# Electroacupuncture for post-stroke dysphagia: A protocol for systematic review and meta-analysis of randomized controlled trials

**DOI:** 10.1097/MD.0000000000022360

**Published:** 2020-09-18

**Authors:** Chang-Ho Han, Jeong Hwa Kim, Mikyung Kim, Ha-Ri Kim, Seo Young Kim, Hyun-Young Choi, Chul Jin, Seungwon Kwon, Woo-Sang Jung, Sang-Kwan Moon, Jung-Mi Park, Chang-Nam Ko, Seung-Yeon Cho

**Affiliations:** a Department of Internal Medicine, College of Korean Medicine, Dongguk University, Gyeongju; b Department of Clinical Korean Medicine, Graduate School, Kyung Hee University, Seoul; c Department of Internal Medicine, College of Korean Medicine, Sangji University, Wonju; d Department of Cardiology and Neurology, College of Korean Medicine, Kyung Hee University, Seoul, Republic of Korea.

**Keywords:** electroacupuncture, post-stroke dysphagia, protocol, stroke, systematic review

## Abstract

**Background:**

Post-stroke dysphagia (PSD) requires effective treatment as it may cause aspiration pneumonia, dehydration, or malnutritution, which can increase the length of hospital stay as well as mortality. In the field of stroke, electroacupuncture (EA) has been widely used, and a number of clinical research papers have been published regarding its effects. This systematic review aims to evaluate the effectiveness of EA for the treatment of PSD.

**Methods:**

Randomized controlled trials evaluating the use of EA in PSD will be included in this meta-analysis. The following electronic databases will be searched from inception to July 31, 2020, using terms relating to EA and PSD: PubMed, the Cochrane Library, the Excerpta Medica Database, China National Knowledge Infrastructure, the Korean Medical Database, KoreaMed, the National Digital Science Library, and the Oriental Medicine Advanced Searching Integrated System. Two reviewers will independently search these databases, select studies for inclusion, and evaluate the quality of the studies. Methodological quality will be assessed using the Cochrane Handbook for Systematic Reviews of Interventions (version 6.0). The primary outcome will be the total effective rate; secondary outcomes will include results of other assessments of dysphagia such as the water drinking test scale and videofluoroscopic swallowing study. We will also investigate the number and severity of adverse events. The Cochrane Review Manager (RevMan) software (version 5.3.5) will be employed to assess bias risk, data integration risk, and meta-analysis risk. Mean difference and standardized mean difference will be used to represent continuous data, while risk ratios will be used for pooled binary data.

**Results:**

This study will provide a comprehensive review and evaluation of the available evidence regarding the efficacy and safety of EA as a treatment for PSD.

**Conclusion:**

This study will clarify whether EA could be an effective and safe treatment for PSD.

## 1 Introduction

Although mortality rates after stroke have decreased due to advances in the initial management of acute stroke (e.g., thrombolysis, mechanical thrombectomy, and hemicraniectomy) and the management of risk factors such as hypertension, diabetes mellitus, and dyslipidemia, neurological and medical complications still result in relatively high morbidity and mortality. Approximately 40% of stroke survivors are classified as disabled (scoring 3–5 on the modified Rankin Scale for between 1 month and 5 years after stroke, therefore, the global burden of stroke remains high.^[1]^ While significant progress has been made in terms of the acute treatment of stroke, the management of sequelae still depends on rehabilitation for each symptom, and even when appropriate rehabilitation therapy is performed, several symptoms often persist for many years.

It has been reported that dysphagia, a common complication of stroke, occurs in 20% to 65% of stroke patients,^[2]^; a further study has found that dysphagia symptoms persist in approximately 50% of patients after discharge from hospital.^[3]^ Post-stroke dysphagia (PSD) requires effective treatment as it may cause aspiration pneumonia, dehydration, or malnutrition, which can increase the length of hospital stay as well as mortality. A variety of interventions, including electrical or magnetic stimulation and behavioral interventions are used in clinical practice. However, the existing evidence from clinical trials and meta-analyses is insufficient to demonstrate the efficacy of these treatments for PSD.^[3]^ Therefore, it is necessary to consider other treatments, including alternative and complementary medical treatments, and to determine their efficacy.

Acupuncture, which is widely used globally, has been recommended by the World Health Organization for stroke treatment and for improving the care of stroke patients.^[4]^ A number of clinical studies have demonstrated the efficacy of acupuncture in stroke patients, and the mechanisms underlying its effect have also been determined.^[4,5]^ Electroacupuncture (EA) is a technique in which an electric current is passed through a pair of inserted acupuncture needles; this method aims to increase the effectiveness of acupuncture by combining it with continuous electrical stimulation. The electrical stimulation used in EA has 3 conditions that can be varied: frequency, power, and the duration of needle retention.^[6]^ These conditions are important determinants of the effect of EA and can be adjusted for different patients depending on the disease and symptoms. In the field of stroke, EA has been widely used, and a number of clinical research papers have been published regarding its effects. However, there are no published systematic review of the evidence regarding EA, especially for the treatment of PSD.^[6,7]^ Therefore, this study aims to evaluate the effectiveness of EA in the treatment of PSD through a systematic review and meta-analysis.

## 2 Methods

### 2.1 Study registration

The protocol for this systematic review has been registered in the ResearchRegistry on August 12, 2020 (reviewregistry961). The review will be conducted in accordance with the preferred reporting item for systematic reviews and meta-analysis protocols guidelines and the Cochrane Handbook for Systematic Reviews of Interventions.^[8–10]^

### 2.2 Inclusion criteria for study selection

#### 2.2.1 Types of studies

This study will only include randomized controlled trials that compare a group treated with EA to a control group. There will be no restrictions regarding the language of publication. Non-randomized controlled trials, quasi-randomized trials, case reports, case series, uncontrolled trials, and laboratory studies will be excluded.

#### 2.2.2 Participants

Only patients diagnosed with PSD will be considered eligible for this study. Recognized clinical diagnostic methods including the videofluoroscopic swallowing study will be used for the diagnosis of dysphagia. There will be no restrictions regarding age, sex, ethnicity, symptom severity, disease duration, length of treatment period, and clinical setting.

#### 2.2.3 Interventions

We will include studies that use EA as an experimental intervention. In this review, we define EA as a procedure in which electrical stimulation is applied to acupuncture needles after they are inserted at acupoints. Studies in which patients in the experimental group were treated with conventional medicine (conventional drugs or rehabilitation therapy) at any frequency, intensity, or duration alongside EA will be included. Studies in which EA was used alongside manual acupuncture will also be included. However, studies will be excluded if they did not aim to verify the effectiveness of EA, even if EA was used as an intervention. Studies that only used manual acupuncture as an experimental intervention will be excluded.

The control interventions will include conventional pharmaceutical drugs, rehabilitation therapy, placebo (sham EA), and traditional Chinese or Korean Medicine therapies other than EA (including manual acupuncture, moxibustion, and cupping therapy). We will exclude studies comparing differences in the frequency or intensity of electrical stimulation, and studies verifying the combined effect of EA and other treatments (e.g., EA plus herbal medicines, EA plus moxibustion, etc). In addition, studies using control interventions that have not been proven to be effective for stroke treatment will also be excluded.

The following treatment comparisons will be investigated:

1. EA vs no treatment
2. EA vs placebo or sham EA
3. EA vs conventional medicine
4. EA+conventional medicine vs only conventional medicine

#### 2.2.4 Outcome measures

To evaluate the effect of treatment on the severity of dysphagia, we will assess the total effective rate as the primary outcome. For secondary outcomes, we will include other measures used to quantify dysphagia such as the water drinking test and videofluoroscopic swallowing study. We will also investigate the number and severity of adverse events.

### 2.3 Data sources

#### 2.3.1 Electronic data sources

The following databases will be searched from inception to July 31, 2020: PubMed, the Cochrane Library, the Excerpta Medica Database, China National Knowledge Infrastructure, the Korean Medical Database, KoreaMed, the National Digital Science Library, and the Oriental Medicine Advanced Searching Integrated System.

#### 2.3.2 Search strategy

The search terms to be used include (“post-stroke dysphagia” or “stroke dysphagia” or “dysphagia after stroke” or “stroke and dysphagia”) and (“electroacupuncture” or “electro-acupuncture”) and (“randomized” or “random”). We will make modifications in accordance with the requirements of different databases, and an equivalent translation of the search terms will be adapted to ensure that similar search terms are used in all databases.

### 2.4 Data collection and analysis

#### 2.4.1 Selection of studies

Two independent reviewers (JHK and SYC) who have a thorough understanding of the field and have been trained on the review process will independently examine the titles, abstracts, and key words of the studies to determine eligibility. In cases of duplicate publication, we will only select the original. After removing duplicates, the full text of studies will be reviewed. EndNote X9 software (clarivate analytics) will be used. The reasons for excluding studies will be recorded and displayed in a PRISMA flowchart. If there is a disagreement between 2 reviewers in the selection process, it will be resolved by discussion between them, and a third reviewer (CHH) will arbitrate if necessary.

#### 2.4.2 Data extraction and management

Reviewers JHK and SYC will independently extract the data from each selected study and fill out the standard data extraction form, which includes general information such as the first author, publication year, language, sample size, characteristics of participants (e.g., age, sex, stroke type), details of randomization, blinding, interventions, treatment period, outcome measures, and adverse events. In the event of disagreements, Reviewer CHH will arbitrate.

#### 2.4.3 Assessment of the risk of bias and the reporting quality of studies

Reviewers JHK and SYC will assess the risk of bias using the Cochrane Collaboration's risk of bias tool. The risk of bias will be judged as “low”, “high”, or “some concerns” for each of the following domains; random sequence generation (selection bias), allocation concealment (selection bias), blinding of participants and personnel (performance bias), blinding of outcome assessment (detection bias), incomplete outcome data (attribution bias), selective outcome reporting (reporting bias), and other sources (other sources of bias). If there are inconsistencies between the judgements of reviewers JHK and SYC, the final decision will be made by reviewer CHH. Methodological quality will be assessed using the Cochrane Handbook for Systematic Reviews of Interventions (version 6.0).

#### 2.4.4 Assessment of heterogeneity

We will perform the *I*^*2*^ test to evaluate statistical heterogeneity. If *I*^*2*^ is >50%, statistical heterogeneity will be considered to be significant and a random-effects models will be used.

#### 2.4.5 Measurement of treatment effect

If there is no heterogeneity, pooled results for continuous data will be presented as the mean difference or standardized mean difference with a 95% confidence interval. If significant heterogeneity is found, a random effects model will be used. For dichotomous data, pooled results will be presented as risk ratios with 95% confidence intervals.

#### 2.4.6 Managing missing data

If there is any missing, incomplete, or unclear data, we will contact the corresponding author to gather extra data as necessary. If this data cannot be obtained, only the data available will be analyzed.

#### 2.4.7 Data synthesis

We will use the Cochrane Review Manager (RevMan) software (version 5.3.5; The Nordic Cochrane Centre, Copenhagen, Denmark) to perform all statistical analyses. If *I*^*2*^ ≤50%, the fixed-effect model will be employed to evaluate the outcome data. Otherwise, the random effects model will be applied. The studies will be synthesized according to the type of intervention and/or control.

We will use software based on the grading of recommendations assessment, development and evaluation framework for Cochrane Systematic Reviews (grading of recommendations assessment, development and evaluation pro guideline development tool software, McMaster University).

#### 2.4.8 Subgroup analysis

If enough studies are available to investigate the factors associated with heterogeneity, subgroup analysis will be conducted.

#### 2.4.9 Sensitivity analysis

We will perform a sensitivity analysis to verify the robustness of the study results. This will be achieved by assessing the impact of sample size, risk of bias, missing data, and statistical models.

#### 2.4.10 Ethics and dissemination

As the data included in this study is not personalized, formal ethical approval is not required for this protocol. We will collect and analyze data based on published studies, and since no patients are directly or specifically assessed in this study, individual privacy will not be a concern. The results of this review will be disseminated to peer-reviewed journals or presented at a relevant conference.

## 3 Discussion

PSD is a common complication of stroke and leads to poor clinical outcomes and high mortality. However, PSD is difficult to treat because it results from damage to the central nervous system.^[2]^ Clinically, a soft diet and feeding tubes are used to prevent aspiration pneumonia resulting from PDS; however, stroke patients with nasogastric tubes in place are known to have a higher risk of death due to respiratory infection. Various non-invasive treatments are used for PSD, such as swallowing rehabilitation by neuromuscular electrical stimulation. However, there is a lack of evidence for the efficacy of these treatments, and further large-scale studies are needed.

In the technique of EA, which combines acupuncture and electrical stimulation, the acupuncture needles can be repeatedly stimulated at a constant frequency and intensity. This technique has been widely used to treat post-stroke symptoms, and the mechanism of its effect has been described in a number of studies.^[11,12]^ EA can be used to treat PSD: several clinical trials have been conducted, but there is still insufficient evidence regarding the efficacy and safety of EA for this condition. The present review aims to assess the efficacy and safety of EA in the treatment of dysphagia after stroke, in order to provide reliable evidence regarding the use of EA to practitioners and patients with PSD.

## Author contributions

**Conceptualization:** Chang-Ho Han.

**Data curation:** Jeong Hwa Kim, Ha-Ri Kim, Seung-Yeon Cho.

**Formal analysis:** Jeong Hwa Kim, Mikyung Kim, Hyun-Young Choi, Seung-Yeon Cho.

**Funding acquisition:** Chang-Ho Han.

**Investigation:** Jeong Hwa Kim, Seo Young Kim, Seung-Yeon Cho.

**Methodology:** Mikyung Kim, Chul Jin, Seungwon Kwon.

**Project administration:** Seung-Yeon Cho.

**Supervision:** Woo-Sang Jung, Sang-Kwan Moon, Jung-Mi Park, Chang-Nam Ko.

**Writing – original draft:** Jeong Hwa Kim.

**Writing – review & editing:** Jung-Mi Park, Seung-Yeon Cho.

## Abbreviations

Abbreviations: EA = electroacupuncture, PSD = post-stroke dysphagia.
